# Bayesian denoising algorithm dealing with colored, non-stationary noise in continuous glucose monitoring timeseries

**DOI:** 10.3389/fbioe.2023.1280233

**Published:** 2023-11-22

**Authors:** Nunzio Camerlingo, Ilaria Siviero, Martina Vettoretti, Giovanni Sparacino, Simone Del Favero, Andrea Facchinetti

**Affiliations:** ^1^ Department of Information Engineering, University of Padova, Padova, Italy; ^2^ Department of Computer Science, University of Verona, Verona, Italy

**Keywords:** Bayesian denoising, continuous glucose monitoring, correlation, stationarity, Butterworth filter

## Abstract

**Introduction:** The retrospective analysis of continuous glucose monitoring (CGM) timeseries can be hampered by colored and non-stationary measurement noise. Here, we introduce a Bayesian denoising (BD) algorithm to address both autocorrelation of measurement noise and temporal variability of its variance.

**Methods:** BD utilizes adaptive, *a-priori* models of signal and noise, whose unknown variances are derived on partially-overlapped CGM windows, via smoothing approach based on linear mean square estimation. The CGM signal and noise variability profiles are then reconstructed using a kernel smoother. BD is first assessed on two simulated datasets, D_S1_ and D_S2_. On D_S1_, the effectiveness of accounting for colored noise is evaluated by comparison against a literature algorithm; on D_S2_, the effectiveness of accounting for the noise variance temporal variability is evaluated by comparison against a Butterworth filter. BD is then evaluated on 15 CGM timeseries measured by the Dexcom G6 (D_R_).

**Results:** On D_S1_, BD allows reducing the root-mean-square-error (RMSE) from 8.10 [6.79–9.24] mg/dL to 6.28 [5.47–7.27] mg/dL (median [IQR]); on D_S2_, RMSE decreases from 6.85 [5.50–8.72] mg/dL to 5.35 [4.48–6.49] mg/dL. On D_R_, BD performs a reasonable tracking of noise variance variability and a satisfactory denoising.

**Discussion:** The new algorithm effectively addresses the nature of CGM measurement error, outperforming existing denoising algorithms.

## 1 Introduction

The use of continuous glucose monitoring (CGM) sensors is rapidly growing in diabetes therapy management and research. This is due to the capability of CGM sensors to provide almost continuous glucose concentration measurements (e.g., every 5 min) over prolonged periods, such as days or weeks (Kumar Das et al., 2022). In the past years, the retrospective analysis of CGM timeseries proved to be important for tuning/refining diabetes therapies and, in turn, for improving the overall glycemic control (Klonoff, 2005; Scheiner, 2016). For example, the offline analysis of trends and patterns in the post-prandial CGM profiles can help optimizing insulin dosages (Battelino et al., 2022), suggesting strategies for taking rescue carbohydrates (Camerlingo et al., 2019), evaluating glucose variability (Service, 2013), quantifying the effectiveness of therapies through, e.g., the computation of time-in-ranges indices (Battelino et al., 2019; Camerlingo et al., 2021), and visualizing clinically-relevant CGM patterns (Danne et al., 2017).

Despite the advancements in sensors technology that have led to increased accuracy, CGM measurements are inevitably impacted by random non-stationary measurement noise, which typically dominates the true signal at high frequencies (Breton and Kovatchev, 2008; Lunn et al., 2011; Laguna et al., 2014; Vettoretti et al., 2019a; Vettoretti et al., 2019b). As a non-stationary random process, the statistical properties of the CGM measurement noise vary over time, or in response to different conditions (both internal mechanisms, such as electromagnetic interferences, electrode degradation, chemical contamination of the sensor surface, and related to the sensor-user interface, such as physical activity, compression, or site insertion inflammation), thus hampering data interpretation.

The signal-to-noise ratio (SNR) in CGM measurements can be improved by utilizing appropriate denoising algorithms. This would enhance the reliability of the clinical conclusions that can be derived from the retrospective analysis of CGM data. To address this issue, a variety of digital filtering and smoothing techniques have been proposed in the literature (Lee et al., 2021; Zhang et al., 2021). Low-pass digital filters, such as Butterworth filters have been often used in the literature (Sparacino et al., 2007; Pérez-Gandía et al., 2010; Mhaskar et al., 2017), as they represent a straightforward way to separate noise bands. However, since signal and noise spectra of CGM measurements normally overlap, it is not possible to remove the random measurement noise without distorting the true glucose values. Kalman filters (Knobbe and Buckingham, 2005; Bequette, 2018), instead, benefit of a maximum likelihood estimation step to determine the parameters of a state space system, modelling the true glucose signal and the measurement noise but, notably, prior knowledge about their statistical properties is needed. In offline setting, the Kalman smoother can be used to get further improvement of the estimates (Staal et al., 2019; Rabby et al., 2021). However, in the above-mentioned methods, the key parameters of the algorithms (e.g., the cutoff frequency for the Butterworth filter) are fixed once for all. This constraint makes such filters unable to adapt their “aggressiveness” to the temporal variations of the CGM measurement error statistical properties (e.g., the variance or the autocorrelation), which have been recently observed on CGM data (Facchinetti et al., 2014; Biagi et al., 2017; Vettoretti et al., 2019b), thus resulting in suboptimal denoised profiles, with oversmoothed or undersmoothed portions.

Despite several adaptive filtering/smoothing algorithms have been proposed in different research fields (Dixit and Nagaria, 2017; Sharma and Pachori, 2018), to the best of our knowledge only few attempts have been made to account for the variability of the SNR in CGM data (Facchinetti et al., 2010; Facchinetti et al., 2011; Zhao et al., 2018; Yadav et al., 2020).

For example, Facchinetti et al. (Facchinetti et al., 2010; Facchinetti et al., 2011) proposed the use of Bayesian estimation approach, where the *a priori* models of measurement error and glucose concentration time course are given by white noise with unknown variance and multiple integration of a white noise with unknown variance, respectively. Both the aforementioned unknown variances can be estimated, with once for all during a burn in interval (Facchinetti et al., 2010), or at specific times (Facchinetti et al., 2011), by using appropriate probabilistic smoothing criteria. Once the values of the models’ variances are determined, the denoised profile can be obtained through linear mean square estimation. While the algorithm of Facchinetti et al. allows to update the filter parameters at user-specified time points, Zhao et al. (2018) proposed later a strategy to update the filter parameters only after a significant change in the noise level, as quantified by an expectation-maximization algorithm, resulting in a significantly shorter computational time. While these adaptive algorithms update the parameters of a state space system to account for the SNR variability, Yadav et al. (2020) proposed an adaptive Savitzky-Golay filter, which can automatically adjust the parameters of a polynomial model (i.e., model order and length of frame), in accordance with the changes in the sampling or cut-off frequency. However, compared to the optimization of the nonparametric models used in (Facchinetti et al., 2010; Facchinetti et al., 2011; Zhao et al., 2018), the optimization of the parametric polynomial model might be computationally expensive, especially if the underlying signal is approximated by a high-order polynomial. Finally, to note, all the above-mentioned algorithms assumed a white Gaussian measurement noise.

Optimality of Bayesian approaches, of course, relies on how accurate the used *a priori* information on the signals into play is. While the expected regularity of glucose time-course is well described by the multiple integration of a white noise (with an unknown variance to be determined from the data), recent research has shown that for certain CGM sensors, the measurement noise cannot be assumed as white, but should be assumed as colored instead. Indeed, a temporal correlation between consecutive samples of CGM measurement noise has been identified in (Biagi et al., 2017; Vettoretti et al., 2019a; Vettoretti et al., 2019b). Specifically, Vettoretti et al. (Vettoretti et al., 2019a) focused on a recent factory-calibrated CGM sensor, the Dexcom G6 (Dexcom, Inc., San Diego, CA), and described its random measurement noise using a second order autoregressive (AR) model, which is consistent with findings reported in (Vettoretti et al., 2019b) for the Dexcom SEVEN Plus (Dexcom, Inc. San Diego, CA) CGM sensor, and in (Biagi et al., 2017) for the Medtronic Paradigm Veo Enlite (Medtronic, Inc., Northridge, CA) CGM sensor. As far as we know, there are no denoising approaches in the literature that can effectively handle colored, non-stationary, measurement error in CGM data. The goal of this paper is to present and evaluate a Bayesian denoising algorithm for retrospective use on CGM data (off-line) which:

1. explicitly accounts for the temporal correlation among measurement noise samples;
2. iteratively estimates the CGM measurement variance in order to adapt its “aggressiveness” to the temporal variability of the SNR.

The proposed algorithm will be thoroughly evaluated against existing algorithms in a simulated environment, where the true underlying signal is known. The performance of the algorithm will be also demonstrated through its application to real-world data, using 15 traces acquired with the Dexcom G6 CGM sensor.

## 2 Materials and methods

### 2.1 The new Bayesian denoising algorithm

In this section, a step-by-step description of the proposed Bayesian Denoising (BD) algorithm is provided. First, a mathematical formulation of the problem is given, and the notations introduced. Then, the prior information used for the Bayesian estimation is documented, and the estimation step is described. Finally, the flowchart of the algorithm is explained.

#### 2.1.1 Problem statement

Let us assume that the CGM measurements are collected in n equally-spaced time instants $kT_{s}$ , with $T_{s}$ being the sampling period and k=1,2,…,n , and that the generic CGM sample $y\left(k\right)$ is given by:

$$y\left(k\right)=u\left(k\right)+w\left(k\right)$$ (1)

where $u\left(k\right)$ is the true (unknown) glucose concentration level, and $w\left(k\right)$ is the measurement noise. Signal and noise are assumed to be mutually uncorrelated.

Let us define the vectors $y={\left[y\left(1\right),y\left(2\right),\ldots ,y\left(n\right)\right]}^{T}$ , $u={\left[u\left(1\right),u\left(2\right),\ldots ,u\left(n\right)\right]}^{T}$ , and $w={\left[w\left(1\right),w\left(2\right),\ldots ,w\left(n\right)\right]}^{T}$ , containing samples of CGM measurements, unknown glucose concentration, and measurement noise, respectively.

In the Bayesian framework, if u and w are zero-mean random vectors with *a priori* covariance matrices denoted by $\Sigma_{u}$ and $\Sigma_{w}$ , respectively, then the linear mean square estimate of u given y is (De Nicolao et al., 1997):

$$\hat{u}={\left(\Sigma_{w}^{-1}+\Sigma_{u}^{-1}\right)}^{-1}\Sigma_{w}^{-1}y$$ (2)

Obtaining the linear mean square estimate thus requires knowledge of the *a priori* covariance matrices $\Sigma_{u}$ and $\Sigma_{w}$ . These can be obtained, in turn, from the corresponding *a priori* model of process variability.

#### 2.1.2 A priori models of signal and noise

As far as the model of $u\left(k\right)$ is concerned, in smoothing/denoising problems, a commonly used approach to describe a regular signal on a uniformly spaced grid is the m-time integration of white noise. For CGM processing purposes, expertise shows that glucose concentration can be well described using m=2 (De Nicolao et al., 1997; Palerm et al., 2005; Mahmoudi et al., 2019), which leads to the so-called integrated random-walk model:

$$u\left(k\right)=2u\left(k-1\right)-u\left(k-2\right)+v\left(k\right)$$ (3)

where $v\left(k\right)$ is a zero-mean Gaussian noise with unknown variance equal to $\lambda^{2}$ .

From Eq. 3, $\Sigma_{u}$ can be easily obtained as:

$$\Sigma_{u}=\lambda^{2}{\left(F^{T}F\right)}^{-1}$$ (4)

where F is the square n -dimensional lower-triangular Toeplitz matrix, whose first column is ${\left[1,-2,1,0,\ldots ,0\right]}^{T}$ . Although very simple, the integrated random-walk model was shown to be able to describe a wide range of signals in a variety of domains, see e.g., (Sparacino et al., 2002; Yue et al., 2020; Kulemann et al., 2021). Its only unknown parameter is $\lambda^{2}$ , which, as shown later in this paragraph, can be estimated from the data through a criterion consistent with the Bayesian embedding.

As far as the CGM measurement error $w\left(k\right)$ of Eq. 1 is concerned, previous works assumed its whiteness, which would bring $\Sigma_{w}$ to be diagonal (Facchinetti et al., 2010). However, for several CGM sensors this assumption is unrealistic, as it has been proven that there is a temporal correlation between consecutive noise samples. To account for it, an AR model of order p can be used:

$$w\left(k\right)=-\sum_{i=1}^{p}a_{i}w\left(k-i\right)+\epsilon \left(k\right)$$ (5)

where $\epsilon \left(k\right)$ is a white noise process with zero-mean and an unknown variance equal to $\sigma^{2}$ . Note that the model of Eq. 5 is general, and the model order p , as well as the values of the model parameters $a_{i},i=1,\ldots ,p$ might be specific to the CGM sensor used. These values need to be identified on a suitable dataset via an appropriate estimation procedure (see, e.g., that proposed in Vettoretti et al. (Vettoretti et al., 2019a)).

From Eq. 5, $\Sigma_{w}$ can be obtained as:

$$\Sigma_{w}=\sigma^{2}{\left(A^{T}A\right)}^{-1}$$ (6)

where A is the square n-dimensional lower-triangular Toeplitz matrix, whose first column is ${\left[1,-a_{1},-a_{2},\ldots ,-a_{p},0,\ldots ,0\right]}^{T}$ . Notably, $\Sigma_{w}$ is no longer diagonal, as it happened in the work by Facchinetti et al. (Facchinetti et al., 2011).

#### 2.1.3 Estimate determination

Once defined $\Sigma_{u}$ and $\Sigma_{w}$ , Eq. 2 turns into:

$$\hat{u}={\left(A^{T}A+\gamma F^{T}F\right)}^{-1}A^{T}Ay$$ (7)

where, as discussed in (De Nicolao et al., 1997), $\gamma =\frac{\sigma^{2}}{\lambda^{2}}$ acts as a parameter which balances the fidelity to the data with the roughness of the estimate, i.e., which regulates the “smoothing aggressiveness”. The value of γ is unknown, because both $\sigma^{2}$ and $\lambda^{2}$ are unknown. Given the inter-individual variability, γ should be personalized. To do so, according to the Bayesian smoothing criteria suggested in (De Nicolao et al., 1997), the problem of Eq. 7 can be solved for several trial values of γ , until the following condition is satisfied:

$$\frac{WRSS\left(\gamma \right)}{n-q\left(\gamma \right)}=\gamma \frac{WESS\left(\gamma \right)}{q\left(\gamma \right)}$$ (8)

where $WRSS\left(\gamma \right)={\left(y-\hat{u}\right)}^{T}A^{T}A\left(y-\hat{u}\right)$ is the weighted residual sum of squares, $WESS\left(\gamma \right)={\hat{u}}^{T}F^{T}F\hat{u}$ is the weighted estimates sum of squares, and $q\left(\gamma \right)=trace\left[\left.A^{T}{\left(A^{T}A+\gamma F^{T}F\right)}^{-1}A\right)\right]$ are the so-called equivalent degrees of freedom. As reported in the aforementioned references, once γ is determined, the estimate $\sigma^{2}$ can be obtained by:

$$\sigma^{2}=\frac{WRSS\left(\gamma \right)}{n-q\left(\gamma \right)}$$ (9)

while the estimate of $\lambda^{2}$ can be obtained by dividing the result of Eq. 9 by γ .

#### 2.1.4 Accounting for non-stationarity of noise

**FIGURE 1:**
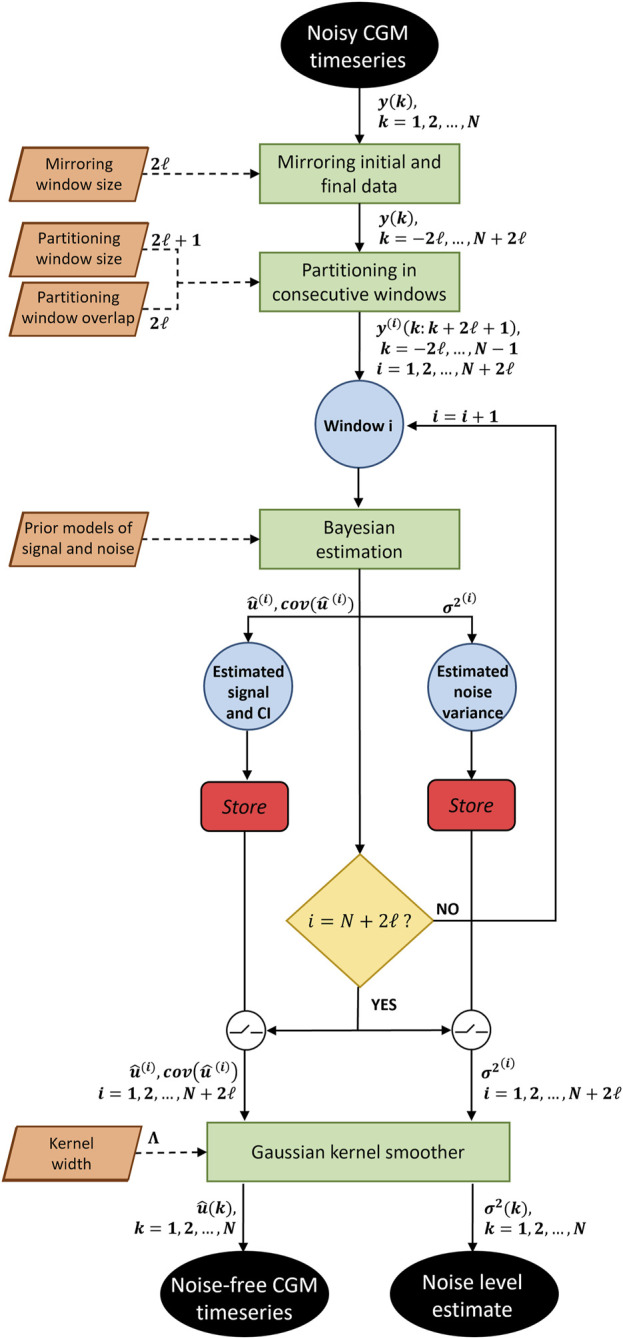
Flowchart of the proposed algorithm. A noisy CGM timeseries is given as input to the algorithm. After mirroring the input data and partitioning it in consecutive windows, a Bayesian estimation is performed for each window, providing the denoised signal with its confidence interval, and the noise variance. Once all windows have been processed, a Gaussian kernel smoother recombine the estimated signals. The algorithm gives as output the noise-free CGM timeseries and the noise level timeseries.

To account for the intra-individual variability of the SNR, the algorithm is applied to consecutive partially-overlapped CGM windows. An optimal “smoothing aggressiveness” is automatically determined for each window, based on the estimated level of noise accounted by γ . Finally, an additional kernel smoother (KS) is put on cascade. A flowchart of the enhanced algorithm is reported in Figure 1, and described step by step below.

Step 1: The algorithm partitions a CGM timeseries into windows of 2l+1 equally-spaced 5-min samples, where neighboring windows overlap by 2l samples.

Step 2: For each window, the mean square estimation is computed, as illustrated in Section 2.1.3. The algorithm estimates $\hat{u}$ as in Eq. 7, and determines the parameters $\sigma^{2}$ and $\lambda^{2}$ (or, similarly γ ) by solving Eq. 8. Note that the parameters of the model describing the CGM measurement error (i.e., the order p , and the coefficients $a_{i},i=1,\ldots ,p$ , of Eq. 5) are assumed to be known and available from the literature (e.g., for the Dexcom G6 sensor can be used those present in Vettoretti et al. (Vettoretti et al., 2019a)). Let us denote the signal estimated from data of window i as ${\hat{u}}^{\left(i\right)}\left(k\right),k=1,2,\ldots ,2l+1,$ and the estimated noise variance as ${\sigma^{2}}^{\left(i\right)}$ . For example, ${\hat{u}}^{\left(1\right)}\left(k\right)$ is obtained by denoising the samples $y\left(k\right)$ , k=1,2,…,2l+1 , while ${\hat{u}}^{\left(2\right)}\left(k\right)$ is obtained considering the samples $y\left(k\right)$ , k=2,3,…,2l+2 .

Step 3: Once noisy data in all windows have been smoothed, the algorithm reconstructs the estimated signal in the overlapped regions by implementing a KS. This strategy effectively eliminates any jumps or discontinuities around the boundaries of neighboring windows. Let us consider a kernel K having the same dimension of the CGM window, i.e., 2l+1 equally-spaced 5-min samples. The final smoothed signal at time c=2l+1,2l+2,…,n−2l is given by the weighted average of the smoothed signals in the neighborhood windows, where the weights are determined by the kernel K :

$$\hat{u}\left(c\right)=\frac{\sum_{i=1}^{2l+1}{\hat{u}}^{\left(c-i+1\right)}\left(i\right)K\left(i\right)}{\sum_{i=1}^{2l+1}K\left(i\right)}$$ (10)

For example, the first point of the smoothed signal $\hat{u}\left(2l+1\right)$ is obtained by weighting the samples ${\hat{u}}^{\left(1\right)}\left(2l+1\right)$ , ${\hat{u}}^{2}\left(2l\right)$ ,…, ${\hat{u}}^{\left(2l+1\right)}\left(1\right)$ .

Step 4: Step 3 is repeated to reconstruct a continuous profile of $\sigma^{2}$ at time c=2l+1,2l+2,…,n−2l, that allows tracking the intra-individual noise variability:

$$\sigma^{2}\left(c\right)=\frac{\sum_{i=1}^{2l+1}{\sigma^{2}}^{\left(c-i+1\right)}K\left(i\right)}{\sum_{i=1}^{2l+1}K\left(i\right)}$$ (11)

Since the signals estimated in windows centered in point c should be weighted more than the signals in the neighbor windows, K was selected as Gaussian kernel centered in c=2l+1,2l+2,…,n−2l , with standard deviation Λ .

The strategy proposed so far does not allow to obtain the final estimates $\hat{u}$ and σ in the very beginning and the very final portions of data of duration 2 l . To overcome this problem, the new algorithm performs the so-called “data mirroring”. The first 2l data samples in y are duplicated, flipped and put before the first sample $y\left(1\right)$ ; similarly, the last 2l data samples in y are duplicated, flipped and put after the last sample $y\left(n\right)$ . Doing so, a sufficient amount of data points (i.e., 2l+1 samples) statistically similar to the actual measurements, are available to feed the KS also at the very beginning/final portions of the CGM timeseries. Once the denoising is completed, the mirrored portions are removed, and the final estimate $\hat{u}$ has the same size ( n ) as the original CGM timeseries y . While a mirroring window size of 2l samples is selected as the minimum size which allows all the y measurements to be denoised, a sensitivity analysis on the mirroring window size has been performed, and described in the Supplementary Material.

The proposed BD algorithm, presents two hyperparameters that need to be set: l , which is the duration of each window the CGM timeseries is partitioned, and Λ , which is the standard deviation of the kernel K . To find suitable values of both parameters, we performed a sensitivity analysis in simulation, testing different values of l ranging in [5:5:25] samples, and different values of Λ ranging in [2:2:14] samples. The values providing the lowest median RMSE over a training set extracted from a synthetic dataset (different from that used to assess the performance of the algorithm) were l=20 samples, and Λ=10 samples. For sake of space, details of this analysis are reported in the Supplementary Material.

### 2.2 Assessment on synthetic and real CGM data

The proposed BD methodology is first assessed on two synthetic datasets of 100 traces each, namely, $D_{S1}$ and $D_{S2}$ , generated using the UVa/Padova simulator (Vettoretti et al., 2018). A great advantage of working in a simulated environment is the availability of both the “ground-truth” noise-free glucose traces, that can be used to assess the denoising effectiveness, and the true value of $\sigma^{2}$ , that can be used to assess the accuracy in the determination of the actual SNR. Note that the UVa/Padova simulator does not generate glucose signals as in Eq. 3, but it leverages numerous differential equations derived from experimental data. Therefore, the structure used to model u (an integrated random walk) could be unsuitable to describe some portions of data with a higher variability (e.g., during rapid glucose rises following meal consumptions), reflecting what eventually happens on real data.

The assessment pipeline implemented therein is incremental. The first dataset $D_{S1}$ simulates CGM data with correlation among noise samples and stationarity of the measurement noise. $D_{S1}$ is used to evaluate the effectiveness of the new BD algorithm to account for the correlation among noise samples. As a comparator, we chose the algorithm of Facchinetti et al. (Facchinetti et al., 2011), which was designed assuming the measurement noise to be white. The second dataset $D_{S2}$ simulates CGM data with correlation among noise samples, but with non-stationary of the measurement noise, which is practically implemented by assuming a temporal variability of the noise variance. $D_{S2}$ is used to evaluate the effectiveness of the new BD algorithm to estimate the variability of the noise variance by adapting the “smoothing aggressiveness” to the SNR. As a comparator, we chose two literature Butterworth filters. For both $D_{S1}$ and $D_{S2}$ , the simulated measurement noise is that of the Dexcom G6 CGM sensor (Dexcom, Inc., San Diego, CA), as it is the only CGM device available on the market with a publicly available statistical model of the measurement noise. Indeed, the order of the AR model in Eq. 5 is p=2 , and the values of $a_{1}$ , and $a_{2}$ are set to the median values reported in Vettoretti et al. (Vettoretti et al., 2019a), and equal to −1.30, and 0.42, respectively.

Finally, the proposed BD methodology is evaluated on the real dataset $D_{R}$ , which consists of 15 CGM traces (extracted from a larger study), collected over adult subjects with type 1 diabetes, wearing a Dexcom G6 CGM sensor (Dexcom, Inc., San Diego, CA) providing 1 sample every 5 min, for up to 10 days. The dataset, courtesy of Dexcom, Inc., is a subset of the dataset collected during the Dexcom G6 Pivotal trial (Wadwa et al., 2018), including 262 participants (53% females, mean ± sd age: 28.0 ± 18.3, diabetes duration: 15.1 ± 13.8, glycated hemoglobin: 8.0% ± 1.3%).

#### 2.2.1 Dataset D_S1_: time-correlated and stationary measurement noise

A set of 100 reference noise-free 1-min sampled glucose profiles of 1-day duration were first generated.

A time-correlated noise profile $w_{1}\left(k\right)$ was then generated as in Eq. 5. To simulate the inter-individual variability of SNR, the value of $\sigma^{2}$ was sampled from a uniform distribution in [4.00–16.00] mg^2^/dL^2^.

**FIGURE 2:**
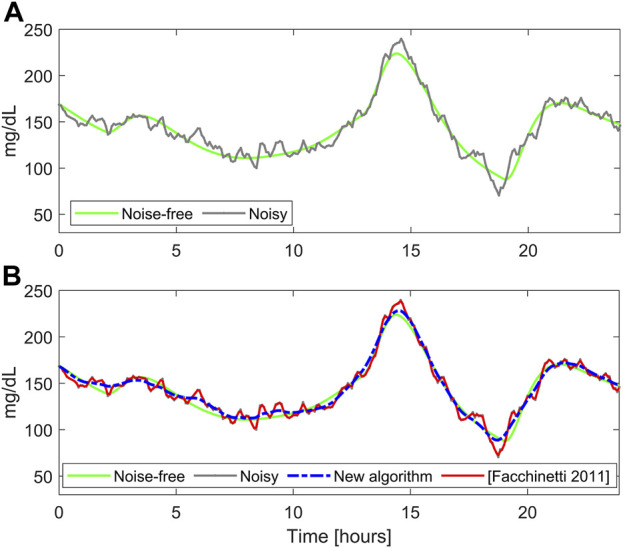
A representative simulated glucose profile with stationary measurement noise. Panel **(A)**: noise-free (green) and noisy CGM data (gray). Panel **(B)** smoothed traces obtained with the literature algorithm (Facchinetti et al., 2011) (red) and BD (blue).

The final synthetic dataset $D_{S1}$ was obtained by adding up the noise-free glucose traces and the noise profiles. Thus, this dataset included 100 glucose traces corrupted by stationary time-correlated measurement noise. A representative profile with $\sigma^{2}$ = 13.33 mg^2^/dL^2^ is shown in Figure 2A (gray curve), together with the noise-free trace (green curve).

The new BD algorithm is compared against the literature algorithm of Facchinetti et al. (Facchinetti et al., 2011), that assumes white measurement noise (i.e., the matrix $\Sigma_{w}$ is diagonal). Besides visualizing the smoothed traces of a representative virtual subject, for each glucose trace of the synthetic dataset, two quantitative performance metrics are calculated: the root-mean-squared-error (RMSE) computed as

$$RMSE=\sqrt{\frac{1}{n}\sum_{i=1}^{n}{\left(\hat{u}\left(i\right)-u\left(i\right)\right)}^{2}},$$ (12)

and the mean-absolute-relative-difference (MARD) computed as

$$MARD=100\cdot \frac{1}{n}\sum_{i=1}^{n}\left|\frac{\hat{u}\left(i\right)-u\left(i\right)}{u\left(i\right)}\right|.$$ (13)

Finally, a Wilcoxon signed rank test with significance level of 1% is performed to test statistical difference among RMSE and MARD distributions.

#### 2.2.2 Dataset D_S2_: time-correlated and non-stationary measurement noise

A set of 100 reference noise-free 1-min sampled glucose profiles of 14-day duration were first generated, as performed for the dataset $D_{S1}$ . Then, a noise profile $w_{2}\left(k\right)$ was generated as in Eq. 5, with $a_{1}=-1.30$ , and $a_{2}=0.42$ , according to (Vettoretti et al., 2019a). Unlike the dataset $D_{S1},\text{to mimic}a$ possible variability of CGM noise variance, $\sigma^{2}$ was modelled as a sinusoid,

$$\sigma^{2}\left(k\right)=A_{0}+A\;\sin\left(\frac{2\pi }{60T}k+\phi \right)$$ (14)

with amplitude A randomly sampled from a uniform distribution in $\left(0,12\right)\;mg^{2}/dL^{2}$ , offset $A_{0}=A+1$ (i.e., in case of $A=10mg^{2}/dL^{2},$ the sinusoid ranges within (1, 21) $mg^{2}/dL^{2}$ ), period T randomly sampled from a uniform distribution in (6, 24) hours, and phase ϕ randomly sampled from a uniform distribution in $\left(-\pi ,\pi \right)$ . These values were selected according to (Facchinetti et al., 2011). However, similar estimation performance was achieved when using different shapes, including square wave and triangular wave.

The final synthetic dataset $D_{S2}$ was obtained by adding up the noise-free traces and the noise profiles. Thus, this dataset included 100 glucose traces corrupted by non-stationary time-correlated noise. A representative glucose profile is shown in Figure 4A, obtained with $\sigma^{2}\left(k\right)=8+7\sin\left(\frac{2\pi k}{12*60}+\pi \right)\;mg^{2}/dL^{2}$ , depicted in Figure 4B.

The new BD algorithm is compared against two literature Butterworth filters: the first is the one used in Sparacino et al. (Sparacino et al., 2007), that will be referred to as BW1, and the second is that employed in Perez-Gandia et al. (Pérez-Gandía et al., 2010), that will be referred to as BW2. Both BW1 and BW2 are first-order low-pass filters, with BW1 more aggressive than BW2 (cutoff frequency normalized to half sampling rate equal to 0.05 for BW1 and 0.1 for BW2). Note that the comparison against the algorithm of Facchinetti et al. (Facchinetti et al., 2011) on $D_{S2}$ has not been reported in this manuscript, as we anticipate that the analysis conducted on $D_{S1}$ will show the superiority of BD when dealing with colored noise.

To evaluate the reliability of BD in denoising the traces of the dataset $D_{S2}$ and in tracking the measurement noise variability, the resulting glucose and $\sigma^{2}$ traces are compared against the “ground-truth” simulated profiles. In addition, to quantitatively assess the performance of the algorithms, RMSE and MARD are computed for each glucose trace of the synthetic dataset $D_{S2}$ , and a Wilcoxon signed rank test is performed to test statistical difference.

## 3 Results

### 3.1 Results on D_S1_

Figure 2B shows the smoothed traces obtained with the literature algorithm (red) and BD (dashed blue), for a representative simulated profile. It is well visible that the literature algorithm performs undersmoothing, reducing only slightly the noise fluctuations, while the new algorithm provides a reliable estimate of the noise-free profile (reported in green). This happens because the literature algorithm underestimates the noise variance ( $\sigma^{2}$ = 2.197 mg^2^/dL^2^), while the new BD allows estimating its value almost perfectly ( $\sigma^{2}$ = 13.26 mg^2^/dL^2^).

Similar considerations can be drawn considering the overall synthetic dataset $D_{S1}$ . The RMSE computed between noisy and noise-free traces is, on median [25th, 75th percentiles], 8.10 [6.79, 9.24] mg/dL, while MARD equals to 4.56 [3.84, 5.29]%. The literature algorithm allows reducing these values only slightly, providing RMSE of 7.89 [6.62, 9.16] mg/dL (*p*-value = 0.02), and MARD of 4.49 [3.79, 5.42]% (*p*-value = 0.06). Instead, BD substantially reduces RMSE to 6.28 [5.47, 7.27] mg/dL (*p*-value<0.0001) and MARD to 3.58 [3.00, 4.26]% (*p*-value<0.0001), thus providing smoothed traces much closer to the “ground-truth” noise-free profiles.

**FIGURE 3:**
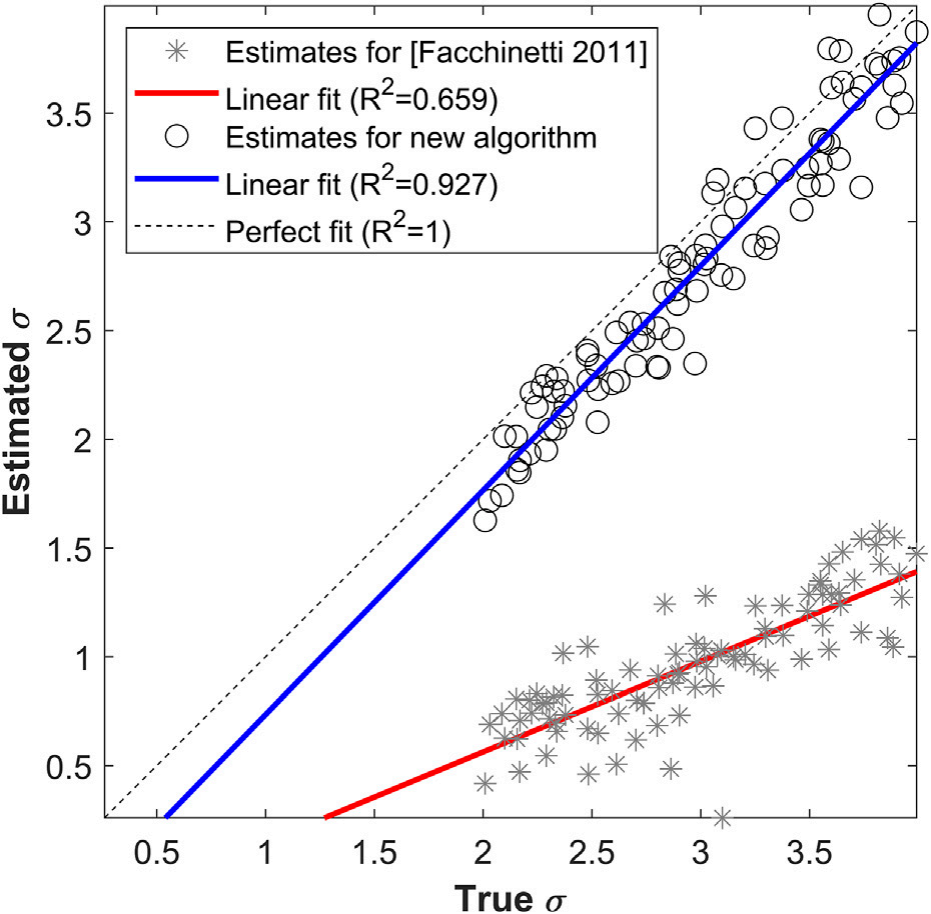
True vs. estimated σ values with the linear fit, for the literature algorithm (Facchinetti et al., 2011) (gray stars and red line) and the new BD algorithm (black circles and blue line). Dashed black line is the bisector, representing the perfect fit.

Figure 3 displays the comparison between true and estimated σ values in the whole dataset $D_{S1}$ , together with the correlation coefficient *R* ^2^, using the literature algorithm (gray stars, with linear fit in red), and BD (black circles, with linear fit in blue). The new algorithm is able to estimate the measurement noise variance accurately (*R* ^2^ = 0.927), notably better than the literature algorithm (*R* ^2^ = 0.659), that substantially underestimates σ , because of its wrong assumption of white measurement noise.

The percent absolute relative error in the estimation of σ is, on median [10th, 90th percentiles], 67.31% [60.22%, 75.19%] for the literature algorithm, 6.74% [2.45%, 14.79%] for the new algorithm. In addition, the estimation of the autoregressive process noise variance $\lambda^{2}$ for the dataset $D_{S1}$ returned an average value of 1.37 mg^2^/dL^2^, with 10th and 90th percentile of 0.25 and 3.83 mg^2^/dL^2^, respectively.

### 3.2 Results on D_S2_

**FIGURE 4:**
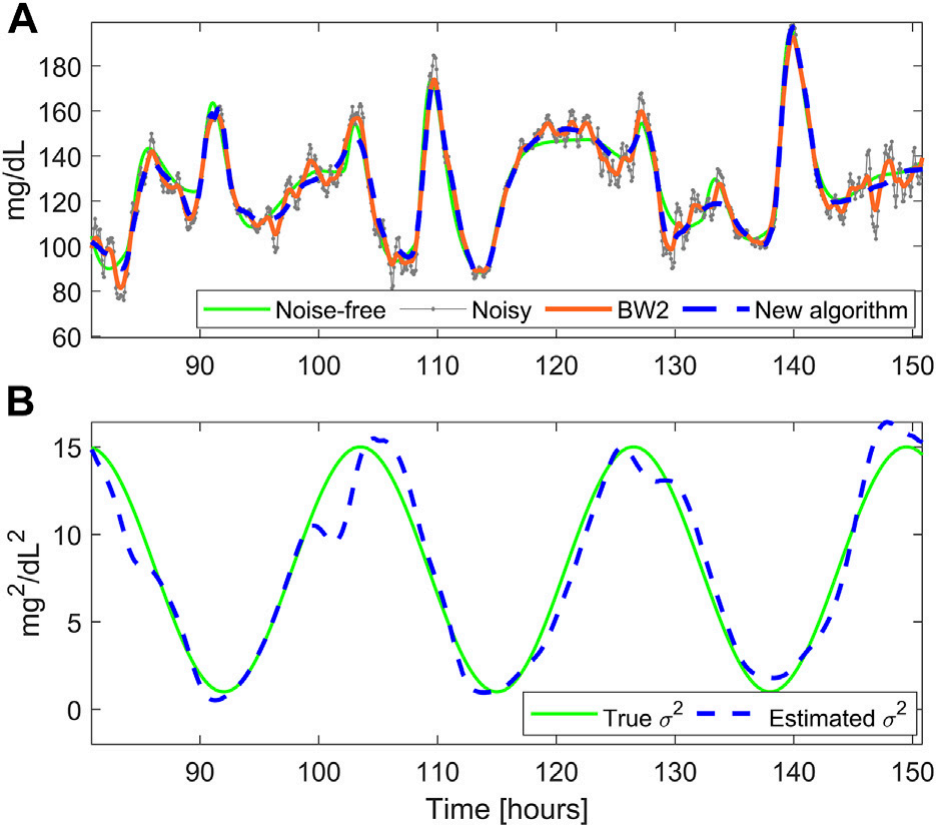
A representative simulated glucose profile with non-stationary measurement noise. Panel **(A)**: noise-free data (green), noisy CGM data (gray), smoothed trace obtained with the new BD (dashed blue), and the Butterworth filter BW2 (orange). Panel **(B)** simulated $\sigma^{2}$ (green) and estimated $\sigma^{2}$ (dashed blue), reflecting the measurement noise variance.

Figure 4A reports the smoothed profile obtained with BD (dashed blue) and the Butterworth filter BW2 (orange solid line). BD performs a satisfactory denoising, being the estimated profile very close to the reference noise-free trace, while BW2 performs undersmoothing for most of the trace. The time course of the estimated $\sigma^{2}$ is reported in Figure 4B, together with the true simulated profile. Notably, the estimated noise variance is very similar to the true one, demonstrating the capability of BD to correctly estimate the intra-individual variability of the SNR.

To quantify the improvement given by BD, in Table 1 we report, for the 100 simulated traces of the dataset $D_{S2}$ , the median [25th, 75th percentiles] values of RMSE and MARD, computed between the “ground-truth” glucose profiles and the smoothed traces obtained with the Butterworth filters BW1 (second column) and BW2 (third column), the algorithm of Facchinetti et al. (fourth column), and the new algorithm (fifth column). In addition, the metrics are computed also between the simulated noisy and noise-free profiles (first column).

**TABLE 1:** RMSE [mg/dL] and MARD [%] computed between the noise-free “ground-truth” profiles and the noisy simulated traces (first column), the traces provided by the filter BW1 (second column), BW2 (third column), and BD (fourth column), for 100 simulated traces.

	Noisy	BW1	BW2	Facchinetti 2011	BD
RMSE	6.85 [5.50–8.72]	8.93 [6.93–10.81]	6.15 [5.22–6.95]	6.77 [5.45–8.62]	5.35 [4.48–6.49]
MARD	3.85 [2.89–4.45]	4.32 [3.58–5.34]	3.26 [2.77–3.86]	3.82 [2.85–4.39]	2.94 [2.29–3.44]

The Butterworth filter BW1 provides higher RMSE and MARD values. This happens because it is too aggressive (i.e., too low cutoff frequency), thus performing oversmoothing in almost all the traces. Instead, the Butterworth filter BW2 provides acceptable RMSE and MARD values. Finally, the new BD algorithm provides the lowest RMSE and MARD values, thus outperforming all comparator algorithms, and resulting significantly different from those provided by the Butterworth filter BW2 (*p*-value<0.0001 for both RMSE and MARD).

**FIGURE 5:**
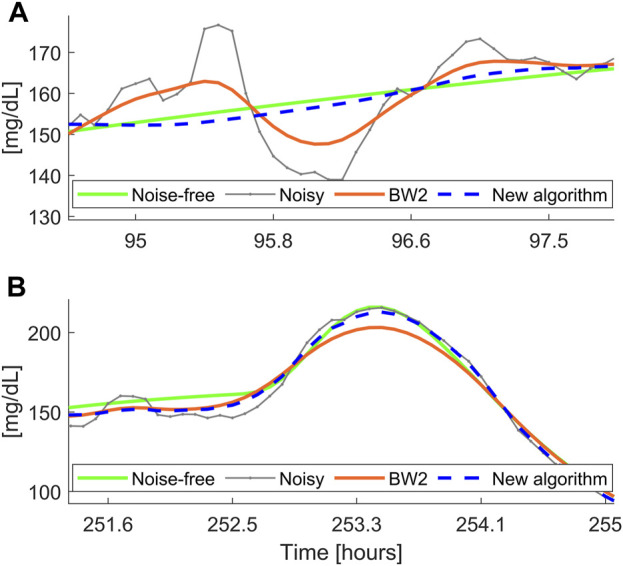
Two representative simulated noise-free (green) and noisy (gray) profiles, with non-stationary measurement noise, filtered with the new algorithm (dashed blue) and the Butterworth filter BW2 (orange). Panel **(A)**: example of undersmoothing of BW2; Panel **(B)**: example of oversmoothing of BW2.

Figure 5 reports a comparison between the smoothed traces obtained with the new BD algorithm and the filter BW2, in two different portions of the dataset $D_{S2}$ . Since BW2 has fixed-parameters, it cannot cope with the intra-individual variability of the SNR. Indeed, it is prone to undersmoothing (in case of low SNR portions), as shown in Figure 5A, and to oversmoothing (in case of high SNR portions), as shown in Figure 5B. On the other hand, in both examples, BD performs a satisfactory denoising, since it is able to adapt its “smoothing aggressiveness” to the estimated SNR.

### 3.3 Results on D_R_

**FIGURE 6:**
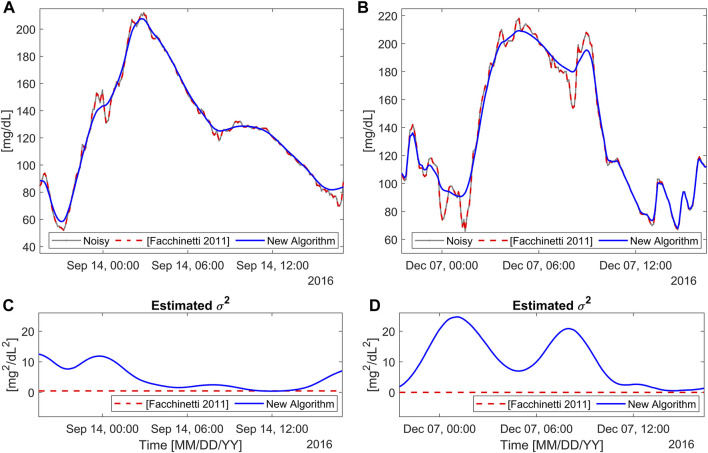
Two representative real profiles from $D_{R}$ . Panels **(A,B)**: CGM data (gray), smoothed trace with the new algorithm (blue), and with the literature algorithm (Facchinetti et al., 2011) (dashed red), for high-SNR [panel **(A)**] and low-SNR [panel **(B)**] profiles. Panels **(C,D)**: estimated $\sigma^{2}$ profiles, reflecting the measurement noise variance, according to the two algorithms.

Figure 6 illustrates the application of the new BD algorithm to two representative portions of the traces of $D_{R}$ : one with a high SNR (left side), and the other with a visibly low SNR (right side). Figures 6A, B display original CGM timeseries (gray), and the smoothed traces obtained with both BD (solid blue) and the literature approach (dashed red) (Facchinetti et al., 2011). As visible by eye inspection, the Dexcom G6 CGM sensor is confirmed to be affected by colored noise, as originally documented in Vettoretti et al. (Vettoretti et al., 2019a). In this situation, the literature algorithm, which assumes that the measurement noise samples are uncorrelated, struggles to effectively filter the noise out. This results in denoised timeseries that are heavily contaminated by noise, with only small and isolated portions of data being acceptably filtered out, in correspondence of the rare instances where the autocorrelation of the measurement noise tends towards zero. Remarkably, the literature algorithm estimates a constant $\sigma^{2}$ , which is proximal to zero, confirming that it is unable to disentangle noise from glucose fluctuations. On the contrary, the CGM timeseries obtained with the proposed BD algorithm are significantly smoother than the original data. The new approach performs a satisfactory denoising in both cases thanks also to the continuous estimation and update of the measurement noise variance, as visible in Figures 6C, D, where the estimated $\sigma^{2}$ profiles are reported. Observing in detail these figures, BD proves to correctly identify a low noise variance for the CGM trace with high-SNR (left panels) and a high noise variance for the CGM trace with low-SNR (right panels), confirming what can be perceived by eye inspection. The estimated noise variance varies in time, confirming the need to cope with the intra-individual variability of SNR. Specifically, in Figure 6C, $\sigma^{2}$ ranges from a minimum of 0.4 mg^2^/dL^2^ to a maximum of 12 mg^2^/dL^2^, with an average of 4.08 mg^2^/dL^2^. In Figure 6D, the estimated $\sigma^{2}$ ranges within 0.6 mg^2^/dL^2^ and 25 mg^2^/dL^2^, with an average of 10.48 mg^2^/dL^2^.

These considerations outlined for the representative data hold for the entire real dataset.

## 4 Limitations

The simulation analysis performed in this paper to assess the performance of the proposed algorithm involved simulated data mimicking the measurement noise corrupting the Dexcom G6 CGM device, as it is the only CGM device available on the market with a publicly available statistical model of the measurement noise. While statistical models of old generation CGM devices are available in the literature (e.g., for the Dexcom SEVEN Plus (Facchinetti et al., 2014), the Medtronic Paradigm Veo Enlite (Biagi et al., 2017), the Dexcom G4 (Facchinetti et al., 2015)), these devices are outdated, and the proposed algorithm should be adapted to cope with other limitations of older technologies, for instance the manual calibration, which would create periodic discontinuities in the CGM traces, affecting the reconstruction of the denoised signal. Statistical models of the measurement noise for other new generation CGM devices (such as Medtronic Guardian Connect, Abbott FreeStyle Libre 3, Ascensia Eversense E3) are not publicly available, and their development would require *ad hoc* datasets with frequently sampled blood glucose values collected in clinic. Although the proposed methodology has not been designed for a specific CGM model, and it is expected to work properly with any colored and non-stationary measurement noise, its assessment on different CGM devices will be performed when the statistical models of their measurement noise will become available.

In addition, the model used in this work assumes the measurement noise to be additive, and signal and noise to be mutually uncorrelated. While these assumptions have been widely used in the literature for biological signals (Facchinetti et al., 2010; Facchinetti et al., 2011; Bequette, 2018; Zhao et al., 2018; Yadav et al., 2020), removing them would require the algorithm to be redesigned.

Finally, the proposed algorithm is retrospective, as it denoises a CGM measurement using data collected before and after that measurement. For this reason, it cannot be applied in real-time. However, in the future we will explore the advantages of implementing the algorithm periodically, for example, every night to denoise the CGM measurements collected in the previous 24 h.

## 5 Conclusion

The retrospective analysis of CGM timeseries is useful to evaluate the effectiveness of diabetes treatments and, in turn, to design personalized therapies. However, the presence of measurement noise may complicate this evaluation. To improve the quality of CGM signals, denoising approaches can be employed. Unfortunately, most approaches exploit digital filters with fixed parameters that cannot cope with neither the inter- nor the intra-individual variability of the SNR in CGM timeseries. In addition, recent investigations showed that, for several CGM sensors, the measurement noise is not only non-stationary, but also colored.

In the present paper we proposed an algorithm which accounts for both autocorrelation and non-stationarity of CGM measurement noise, by leveraging the Bayesian theory. The performance of the new algorithm was assessed on both simulated and real data. Simulation results proved the effectiveness of the method and its superiority versus literature approaches, being effective in correctly estimating the measurement noise variance and tracking its time-variability. Application on real data showed that the measurement noise variance determined by the new algorithm over time was in agreement with the SNR qualitatively perceivable by eye inspection. In summary, the new algorithm can be used to effectively perform retrospective denoising of CGM timeseries, providing a reliable estimate of both the glucose profile and the measurement noise variance time-variable pattern.

Notably, being the proposed algorithm devised in a Bayesian framework, it is theoretically and practically possible to obtain also the confidence interval of the estimated CGM denoised profiles. Future work will explore the use of confidence intervals as a measure of reliability and how this feature could be exploited in practical challenges related to CGM sensors, such as identifying pressure-induces artifacts. Finally, we will also investigate how to adapt the proposed algorithm to smooth different biological signals, such as heart rate or blood pressure, and to quantify the amount of measurement noise present on these timeseries.

In conclusion, we contend that the algorithm presented in this work represents a significant advance in the field of retrospective denoising of CGM timeseries. Indeed, as opposite to the literature algorithms accounting for the non-stationarity of CGM timeseries (Facchinetti et al., 2011; Zhao et al., 2018; Yadav et al., 2020), which assumed the measurement noise to be white, the proposed algorithm represents the first attempt to simultaneously account for both the non-stationarity and the time-correlation of the measurement noise.

## Data Availability

The datasets presented in this study can be found in online repositories. The names of the repository/repositories and accession number(s) can be found below: https://github.com/NunzioCamer/Bayesian-Denoising-CGM.
