# Supplementary material for: Bayesian denoising algorithm dealing with colored, non-stationary noise in continuous glucose monitoring timeseries
Source: Front Bioeng Biotechnol. 2023 Nov 22;11:1280233. doi: 10.3389/fbioe.2023.1280233 (PMC10703295; doi:10.3389/fbioe.2023.1280233)
Supplement: Supplementary file 1 [file DataSheet1.docx]

Supplementary material

Bayesian denoising algorithm dealing with colored, non-stationary noise in continuous glucose monitoring timeseries

Nunzio Camerlingo^1^, Ilaria Siviero^2^, Martina Vettoretti^1^, Giovanni Sparacino^1^, Simone Del Favero^1^, Andrea Facchinetti^1^

^1^Department of Information Engineering, University of Padova, Padova, Italy

^2^Department of Computer Science, University of Verona, Verona, Italy

*** Correspondence:**Andrea Facchinetti
facchine@dei.unipd.it

# A sensitivity analysis to determine suitable values of$\mathcal{l}$ and $\boldsymbol{\Lambda}$

The proposed BD algorithm method presents two hyperparameters that need to be set: $\mathcal{l}$, which is the duration of each window the CGM timeseries is partitioned, and $\Lambda$, which is the standard deviation of the kernel smoother. To find suitable values of both parameters, a sensitivity analysis in simulation was performed.

A simulated dataset D_S3_ consisting of 100 CGM traces was generated by corrupting the noise-free glucose traces (simulated with the UVA/Padova T1D simulator), with colored and non-stationary measurement noise, mimicking that corrupting the Dexcom G6 CGM device, as done to generate the dataset D_S2_.

The dataset has been divided into 70% training set and 30% test set on a subject level. On the training set, suitable hyperparameters have been selected via grid search analysis. Specifically, with $\mathcal{l}$ ranging in [5:5:25] samples, and $\Lambda$ ranging in [2:2:14] samples, a suitable pair of hyperparameters is selected as those minimizing the RMSE.

Supplementary Figure S1 shows the median across the training set subjects of the RMSE between the noise-free simulated CGM profile and the denoised CGM profile, for different window sizes $\mathcal{l}$ (x axis), and different kernel widths $\Lambda$ (color legend). The lowest RMSE values are reported in the red circle, obtained with $\mathcal{l}$ equal to 20 samples, while $\Lambda$ ranging in [10-14] samples. These pairs provide a median RMSE ranging in [5.17, 5.19] mg/dL.

Considering such a limited RMSE variability, we selected the lowest value of $\Lambda$, i.e., $\Lambda=10$ samples, in order to minimize the computational time.

The values of $\mathcal{l}$ =20 samples and $\Lambda=10$ samples led to the following results on the test set (reported as mean [25^th^, 75^th^ percentiles]: RMSE=5.46 [3.95, 6.57] mg/dL, and MARD=2.90 [1.98, 3.50] %.

Notably, in general, the algorithm’s parameters can be selected by the user, based on the available information on the measurement noise corrupting the CGM signal to be filtered.


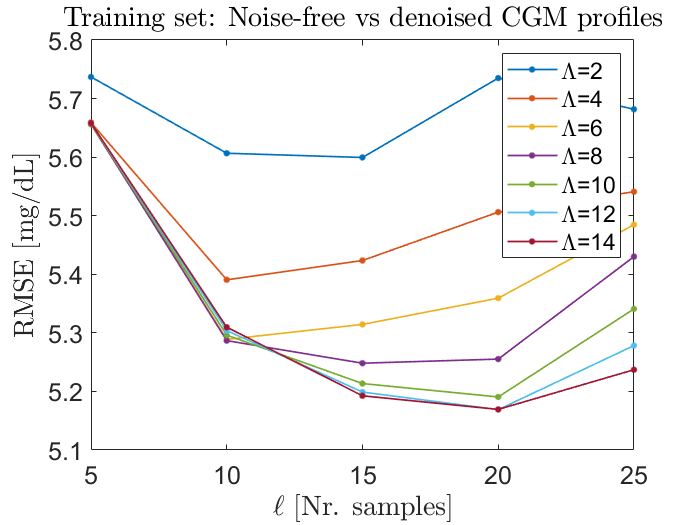


**Supplementary Figure S1.** Median RMSE across 100 simulated CGM traces, for different window sizes $\mathcal{l}$ (x axis), and different kernel widths $\Lambda$ (color legend). The red circle indicates the lower RMSE values.

# A sensitivity analysis over the mirroring window size

The proposed algorithm requires $2\mathcal{l}$ CGM samples before and after each measurement to denoise it. The problem arises for the $2\mathcal{l}$ initial CGM samples and the last $2\mathcal{l}$ CGM samples, since these points do not have sufficient data before and after, respectively, to the denoised. To overcome this problem, we decided to resort to the mirroring approach, which consists in flipping the initial $M$ CGM samples and the last $M$ CGM samples ($M\geq2\mathcal{l}$) to denoise the $2\mathcal{l}$ initial CGM samples and the last $2\mathcal{l}$ CGM samples.

Note that, once the timeseries has been denoised, the extra $2M$ samples at the head and tail are removed, and the RMSE is computed between the denoised profile and the simulated noise-free trace, both of $N$ samples.

To evaluate the possible impact of the mirroring approach, we performed a sensitivity analysis over the dataset D_S3_. The analysis considers different values of $M$, ranging within 40 (i.e., $2\mathcal{l}$ samples in the preferred embodiment of $\mathcal{l=}20$) and 60 samples.

Supplementary Figure S2 shows that the boxplots of RMSE between the denoised profile and the noise-free trace are almost identical, for different values of $M$. As a note, the RMSE values vary between 5.188 and 5.190 mg/dL, for the effect of the kernel smoother applied before removing the extra $2M$ samples.

This analysis suggests that the mirroring approach is useful to denoise the entire CGM timeseries, but the duration of the mirroring window has an almost null impact on the final performance of the algorithm.


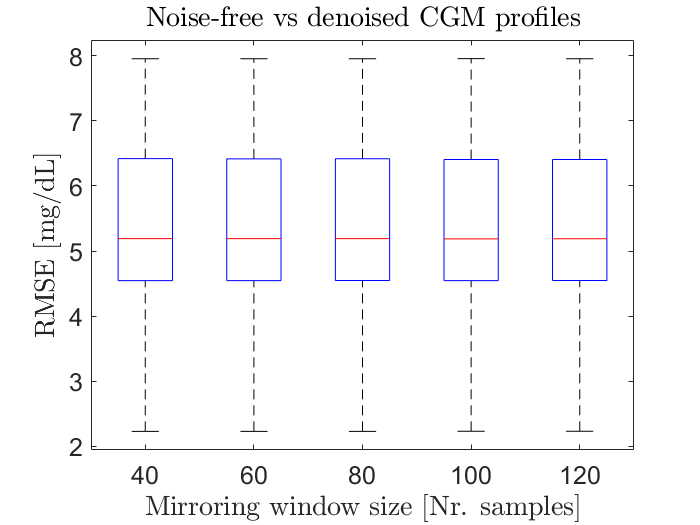


**Supplementary Figure S2.** Boxplots of RMSE across 100 simulated CGM traces, for different mirroring window sizes $M$.
